# Community-Led Total Sanitation Moves the Needle on Ending Open Defecation in Zambia

**DOI:** 10.4269/ajtmh.19-0151

**Published:** 2019-03-11

**Authors:** Joe Brown, Jeff Albert, Dale Whittington

**Affiliations:** 1 School of Civil and Environmental Engineering, Georgia Institute of Technology, Atlanta, Georgia;; 2 Aquaya Institute, San Anselmo, California;; 3 Department of Environmental Sciences and Engineering, University of North Carolina at Chapel Hill, Chapel Hill, North Carolina;; 4 Department of City and Regional Planning, University of North Carolina at Chapel Hill, Chapel Hill, North Carolina;; 5 Global Research Institute, University of Manchester, Manchester, United Kingdom

Community-led total sanitation (CLTS) has emerged as the most widely implemented policy intervention for improving rural sanitation in low-income countries. Community-led total sanitation is focused on the SDG of ending open defecation (OD), still practiced by nearly 900 million people.^1^ Large-scale CLTS programming is underway in dozens of countries and represents an appealing option to governments and donors, promising reductions in OD and increases in sanitation coverage through community mobilization and collective behavior change, typically without direct subsidies for toilet construction. A rich literature on CLTS has emerged documenting a range of programmatic conditions and experiences and an increasingly sophisticated understanding of CLTS’s potential advantages and limitations.^2^

In this issue, Yeboah-Antwi and others describe results from a pre- and post-assessment of national-scale CLTS programming in Zambia,^3^ conducted from 2013 to 2016. Three years after the CLTS intervention, the authors measured a 15.9 percentage point increase in access to improved sanitation facilities, a 4.8 percentage point decline in households lacking access to any toilet, modest increases in handwashing behavior and dedicated hand hygiene spaces, and a 10.3 percentage point increase in households self-reporting that they live in an OD-free village compared with baseline. Despite lacking a control group to measure secular trends in sanitation—the country saw a considerable decline in rural OD in the period from 1990 to 2012, dropping from 42% in 1990 to 25% in 2012^4^—substantial progress in rural sanitation can reasonably be attributed to the intervention. Yeboah-Antwi and others found rural sanitation coverage to increase well beyond the estimated 5% gain in improved sanitation observed nationwide in Zambia during the prior two decades.^4^ The program’s estimated increase in sanitation coverage was consistent with ranges reported in recent systematic reviews^5,6^ of CLTS elsewhere.

Progress in expanding and improving rural sanitation is usually best measured in decades. Logistical, financial, and other constraints mean that promising approaches—even ones with transformative potential at local scales—require enough time, investment, and sectoral support to meaningfully increase access to sanitation and improve overall water, sanitation, and hygiene conditions. As with many paradigm-shifting approaches intended to solve complex intractable problems, early enthusiasm for CLTS has been tempered by experience in taking the intervention to scale in diverse contexts. As the method matures and more evidence from the field is accumulated and synthesized, performance will hopefully improve. Community-led total sanitation implementation protocols continue to evolve as more data become available and programs are modified to suit local needs and capacities. Vigorous debate continues on such topics as the role of subsidies, appropriateness of different modalities for achieving sustained behavior change, and the potential for translating increases in community sanitation coverage into health impacts. Based on current unknowns, we identify a number of priorities for continuing research in CLTS.

First, implementation research may allow for further refinement of CLTS methodology and associated programming. Although CLTS can be successful in ending OD at the level of individual villages, sometimes quite rapidly, progress at scale can be inconsistent, slow, and may not be sustained. Continued long-term engagement with communities is often needed, and overall gains in sanitation coverage or other outcomes are uneven.^5^ A number of variables have been identified as likely associated with CLTS success, including various criteria for community selection (e.g., lack of previous subsidy programs and current environmental and social/cultural conditions),^7^ intensity and duration of follow-up, involvement of skilled and motivated leaders,^8^ social cohesion, and community participation.^9^ Although CLTS is unlikely ever to be a “one-size-fits-all” solution, efforts to further refine the model should allow for smarter targeting of resources to achieve impact.

Second, given that CLTS is intended to facilitate the containment of human excreta and reduce potential for exposure to enteric pathogens, questions remain on whether and how the quality, durability, use, and function (in terms of fecal waste containment) of latrines can be influenced via adjunct programming or an enabling environment. New latrine designs, accompaniment of sanitation marketing,^10,11^ supply chain development, and technical support to communities may help ensure that what gets built is likely to result in reduced exposure to those most at risk, and unlikely to be abandoned. The application of targeted subsidies in the context of CLTS—long considered to be at odds with the approach—may be helpful in this regard, in addition to helping reach the poorest,^12,13^ who may be least likely to construct latrines.

Third, the contention that safe and reliable sequestration of human excreta can interrupt the transmission of pathogens is uncontroversial. Pathogens in feces cannot be transmitted to new hosts unless there are opportunities for direct or indirect contact with fecal waste. But the evidence base for sanitation generally and CLTS specifically to reliably deliver reductions in diarrheal diseases or positively impact other more distal outcomes such as growth and development has never been more debated.^14^ Despite systematic reviews of sanitation suggesting reductions in diarrhea^14^ and impacts on other outcomes,^15^ several recent large, rigorous, controlled trials of rural sanitation showed no effect on most outcomes^16–20^; one showed a reduction in diarrheal prevalence from 5.7% to 3.5%.^20^ With respect to CLTS and CLTS-like interventions specifically, three controlled trials reported an impact on child growth^21–23^ (one of these with a marginal effect on diarrhea) and another showed a reduction in prevalence of roundworm infection^9^ (but no effect on either diarrhea or growth). At least six trials found no health effects of CLTS.^6^ Numerous factors may limit attempts to synthesize these disparate findings, including underlying heterogeneity in trial contexts; interventions (particularly the role of community-level focus); baseline coverage and changes in coverage, time, and behaviors; enteric infections; and routes of transmission.^24–26^ There is reason to believe that achieving complete or near-complete coverage of effective sanitation can yield so-called herd-protective effects,^27–29^ but a demonstration of CLTS’s ability to consistently produce them remains elusive.

More broadly, it is becoming clear that CLTS and similar interventions—as they are currently implemented, at scale—cannot be expected to always or even usually impact diarrhea, stunting, and related outcomes that have been the focus of recent health trials, undermining the case^30,31^ for their adoption. If clinical trials of a new pharmaceutical drug had results like those currently available for CLTS, the U.S. Food and Drug Administration would not approve the drug. Following findings of no effect in recent health impact trials, researchers have argued that a sanitation intervention “may still be valuable as it has other social benefits”^17^ and that null findings “should not diminish ongoing, ambitious efforts to achieve the UN SDGs: myriad health, equity, and ethical arguments motivate elimination of OD and ample supply of microbiologically safe water, even in the absence of a strong link to child growth.”^32^ Other health and non-health benefits of sanitation may or may not be sufficient to justify the considerable cost of these programs; one needs to compare the benefits and costs to find out. Such benefits should thus be identified and incorporated into future trials. Future research may reveal opportunities to develop better sanitation programming and to pursue transformative interventions to interrupt transmission of enteric pathogens. Sanitation sector professionals should adjust their expectations about what CLTS can realistically deliver in terms of at-scale health gains over time scales of controlled trials research, and be prepared to rethink the value proposition of rural sanitation initiatives as new evidence becomes available.
